# A Case of Prostatic Signet-Ring Cell-like Carcinoma with Pagetoid Spread and Intraductal Carcinoma and Long-Term Survival: PD-L1 and Mismatch Repair System Proteins (MMR) Immunohistochemical Evaluation with Systematic Literature Review

**DOI:** 10.3390/jpm13061016

**Published:** 2023-06-19

**Authors:** Nektarios Koufopoulos, Argyro-Ioanna Ieronimaki, Andriani Zacharatou, Alina Roxana Gouloumis, Danai Leventakou, Ioannis Boutas, Dionysios T. Dimas, Adamantia Kontogeorgi, Kyparissia Sitara, Lubna Khaldi, Magda Zanelli, Andrea Palicelli

**Affiliations:** 1 Second Department of Pathology, Medical School, National and Kapodistrian University of Athens, Attikon University Hospital, 15772 Athens, Greece; 2 Breast Unit, Rea Maternity Hospital, P. Faliro, 17564 Athens, Greece; 3 Breast Unit, Athens Medical Center, Psychiko Clinic, 11525 Athens, Greece; 4 Third Department of Obstetrics and Gynecology, Medical School, National and Kapodistrian University of Athens, Attikon University Hospital, 15772 Athens, Greece; 5 Department of Internal Medicine, “Elpis” General Hospital of Athens, 11522 Athens, Greece; 6 Pathology Department “Saint Savvas” Anti-Cancer Hospital, 10447 Athens, Greece; 7 Pathology Unit, Azienda USL-IRCCS di Reggio Emilia, 42123 Reggio Emilia, Italy

**Keywords:** prostatic carcinoma, signet ring cell carcinoma, intraductal carcinoma, pagetoid spread, metastasis, PD-L1, mismatch repair system

## Abstract

Prostatic adenocarcinoma (PA) is the second most common malignancy in men globally. Signet-ring cell-like adenocarcinoma (SRCC) is a very rare PA subtype, with around 200 cases reported in the English literature. Histologically, the tumor cells show a vacuole compressing the nucleus to the periphery. Pagetoid spread in acini and ducts is usually related to metastases from urothelial or colorectal carcinomas, less commonly associated with intraductal carcinoma (IC); histologically, the tumor cells grow between the acinar secretory and basal cell layers. To our knowledge, we report the first prostatic SRCC (Gleason score 10, stage pT3b) associated with IC and pagetoid spread to prostatic acini and seminal vesicles. To our systematic literature review (PRISMA guidelines), it is the first tested case for both PD-L1 (<1% of positive tumor cells, clone 22C3) and mismatch repair system proteins (MMR) (MLH1+/MSH2+/PMS2+/MSH6+). We found no SRCC previously tested for MMR, while only four previous cases showed high expression of another PD-L1 clone (28-8). Finally, we discussed the differential diagnoses of prostatic SRCC.

## 1. Introduction

Adenocarcinoma of the prostate (PA) is the second most common cancer and the fifth leading cause of mortality among men; about 1.4 million newly diagnosed cases were estimated worldwide in 2020 [1]. Acinar adenocarcinoma is the most frequent histotype of PA, while the signet-ring cell-like adenocarcinoma of the prostate (SRCC) is a rare entity that accounts for about 2.5% of PA patients. It is frequently combined with more conventional acinar-type areas. SRCC was first reported in 1981. Around 200 cases have been described in the English literature since then [2,3,4,5]. Histologically, the signet ring morphology of the tumor cells is characterized by an intracytoplasmic vacuole that compresses the nucleus into a crescent shape, displacing it to the cell periphery [6,7].

Signet-ring cell carcinomas are more common in the stomach (3–4% of all gastric cancers) [8], followed by colon, pancreas, breast, thyroid, bladder, and prostate [9]. Moreover, several other tumors can show a signet-ring-like cell appearance, including smooth muscle tumors [10], mesotheliomas [11], oligodendrogliomas [12], thyroid tumors [13,14], ovarian tumors [15], and lymphomas [16]. So, the diagnosis of a primary prostatic SRCC requires a multidisciplinary approach, including histopathological evaluation, gastrointestinal endoscopic examination, and radiologic assessment to exclude metastatic involvement of the prostate [17,18].

Intraductal carcinoma (IC) of the prostate is characterized by an in situ proliferation of malignant cells showing high-grade nuclear atypia within pre-existing acini and prostatic ducts. Being reported in 15–31% of routinely processed radical prostatectomies, it is frequently associated with an invasive high-grade prostatic carcinoma showing advanced clinical stage, larger tumor volume, and poor prognosis [19].

Pagetoid spread (PS) is the growth of cells between the secretory and basal cell layers of the acini [20]. PS in the prostate is usually associated with metastasis from urothelial or colorectal carcinomas and is rarely associated with IC or SRCC [20].

Pembrolizumab is a monoclonal anti-PD-1 antibody and an immune checkpoint inhibitor (ICI) that binds with high affinity to PD-1, a cell surface receptor expressed by inflammatory cells (activated T, NK, B cells, and monocytes). The interaction of PD-1 with its ligand PD-L1 (sometimes expressed by tumor cells) can inhibit the host anti-tumor immune response. In some tumor types, the immunohistochemical evaluation (IHC) of PD-L1 and/or mismatch repair system proteins (MMR) (MSH2, MSH6, MLH1, and PMS2) expression is recommended to allow ICI administration, which can favor the anti-tumor immune response; combined MMR/PD-L1 testing was rarely performed in PA [21,22,23,24,25,26,27,28,29,30,31,32,33,34].

We herewith report a case of SRCC of the prostate (stage pT3b, Gleason score 10) associated with IC, revealing PS to the adjacent prostatic acini and seminal vesicles and showing long-term survival. To our knowledge, this is the first case of prostatic SRCC associated with IC and PS in the English literature. We also tested MMR and PD-L1 by IHC in our case. We also performed a systematic literature review of the expression of these markers in prostatic SRCCs; no relevant data concerning combined MMR/PD-L1 testing were previously reported. Finally, we discussed the differential diagnosis of SRCC of the prostate.

## 2. Materials and Methods

### 2.1. Our Case

The biopsy/surgical specimens were fixed in 10% buffered formalin and routinely processed. Formalin-fixed, paraffin-embedded blocks were sectioned, and the histological slides were stained with hematoxylin–eosin and immunohistochemical markers. We employed the following antibodies: PSA (PSA mouse monoclonal ER-PR8, Zytomed Systems, Berlin, Germany) and P504S (AMACR rabbit monoclonal 13H4, DAKO, Carpinteria, CA, USA), CDX-2 (CDX2 rabbit monoclonal EPR2764Y, Zytomed), CK7 (cytokeratin-7 mouse monoclonal OV-TL, Zytomed), CK20 (cytokeratin-20 mouse monoclonal Ks20.8, DAKO), SMA (Alpha-Smooth Muscle Actin mouse monoclonal 1A4, DAKO), and LCA (CD45, LCA mouse monoclonal 2B11/PD7/26, DAKO) 13H4, DAKO), GATA-3 (GATA 3 mouse monoclonal L50-823, Zytomed), and CK34βE12 (HMWCK mouse monoclonal 34bE12, Zytomed).

### 2.2. Systematic Literature Review

**Figure 1:**
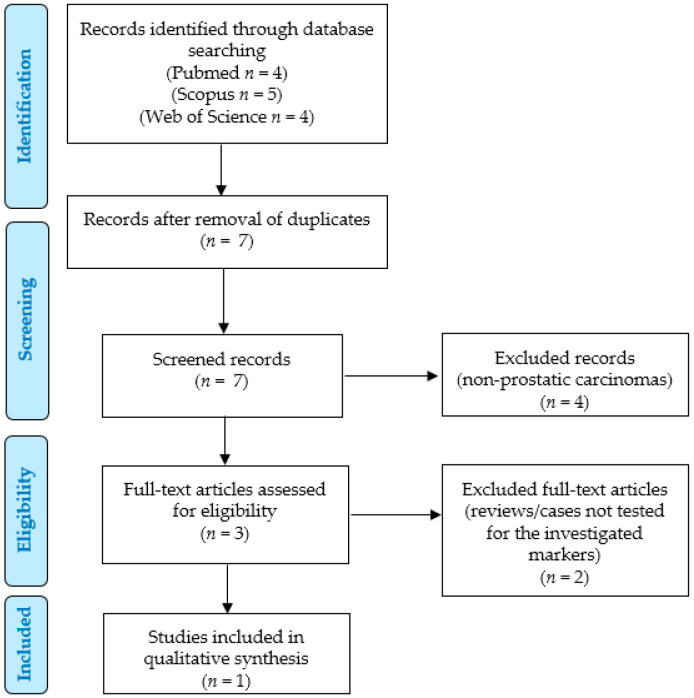
Systematic literature review: PRISMA flow chart.

We performed a systematic review of the literature according to the “Preferred Reporting Items for Systematic Reviews and Meta-Analyses” (PRISMA) guidelines (http://www.prisma-statement.org/; accessed on 1 May 2023) (Figure 1) to identify the previously reported cases of primary prostatic SRCC that had been evaluated for PD-L1 or MMR expression.

Our retrospective observational study was conducted through the PICO process:

- Population: men with a diagnosis of primary prostatic SRCC;
- Intervention: any;
- Comparison: none;
- Outcomes: patients’ clinical outcomes (survival and recurrence rates, status at last follow-up).

We searched for ((prostate OR prostatic) AND (carcinoma OR carcinomas OR adenocarcinoma OR adenocarcinomas OR cancer) AND (“signet-ring” OR “signet ring”) AND (mismatch OR MLH-1 OR MLH1 OR MSH2 OR MSH-2 OR PMS-2 OR PMS2 OR MSH6 OR MSH-6 OR MMR OR d-MMR OR dMMR OR pMMR OR p-MMR OR microsatellite OR MSI OR MSS OR MSI-H OR MSI-L OR MSI-high OR MSI-low OR PD-L1 OR CD274)) in the Pubmed (all fields; 4 results; https://pubmed.ncbi.nlm.nih.gov, accessed on 1 May 2023), Scopus (Title/Abstract/Keywords; 5 results; https://www.scopus.com/home.uri, accessed on 1 May 2023) and Web of Science (all fields; 4 results; https://login.webofknowledge.com, accessed on 1 May 2023) databases. No limitations or additional filters were set. The bibliographic research ended on 1 May 2023. We applied the following criteria:

- Eligibility/inclusion criteria: studies reporting human cases of primary prostatic SRCCs tested for PD-L1, mismatch repair system proteins, and/or microsatellite instability by IHC or molecular assays.
- Exclusion criteria: unclear diagnosis; signet ring cell carcinomas originating from other sites; non-analyzable results (too aggregated data); review articles; cases not tested for the investigated markers.

Two independent authors removed the duplicates and checked the titles and abstracts of all the retrieved results (*n* = 7). After applying the eligibility, inclusion, and exclusion criteria, they selected 3 relevant eligible papers, which were all obtained in full-text format and screened for additional references. After reading the full texts, 2 articles were excluded for being unfit according to the inclusion/exclusion criteria (review articles; cases not tested for the investigated markers). Data collection was study- and case-related. As only 1 paper (describing 4 cases) was finally included [2], no significant statistical analysis was performed; the details of the article were reported in the Section 4.

## 3. Case Report

**Figure 2:**
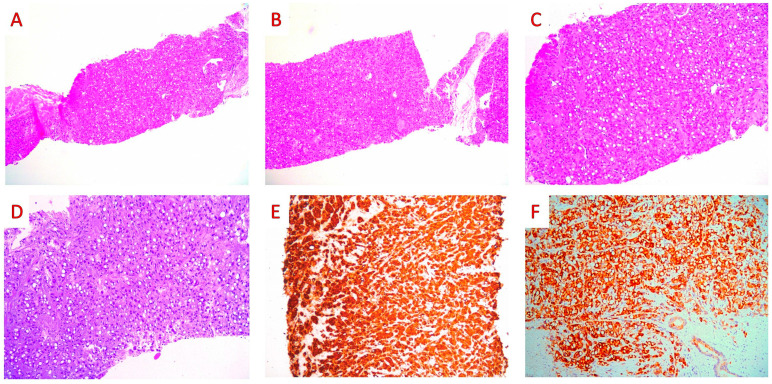
Core biopsies: histopathologic findings. (**A**,**B**) On low-power examination, the core biopsy is infiltrated by a high-grade adenocarcinoma arranged mainly in small nests (hematoxylin and eosin, H&E; ×100). (**C**,**D**) On higher-power examination, the nucleus of several tumor cells is displaced to the periphery, revealing signet ring morphology (H&E; ×200). (**E**,**F**) On immunohistochemical examination, the cytoplasm of the tumor cells stained for PSA (PSA mouse monoclonal ER-PR8, Zytomed; ×200) and P504S (AMACR rabbit monoclonal 13H4, DAKO; ×100) (original, previously unpublished photos).

A 63-year-old patient was admitted to our hospital due to acute urinary retention and hematuria. The prostate was enlarged, irregular, and hard on digital rectal examination. Serum prostate-specific antigen (PSA) levels were elevated (16.39 ng/mL). A transrectal ultrasound-guided mapping biopsy was performed. Cell clusters of high-grade adenocarcinoma infiltrated all twelve (6 from each lobe) of the tissue cores with a signet ring cell component accounting for around 50% of the tumor volume. Foci of intraductal carcinoma or prostatic intraepithelial neoplasm (PIN) were absent. On immunohistochemical examination, the tumor cells were positive for PSA and P504S, being negative for CDX-2, CK7, CK20, SMA, and LCA (Figure 2).

The pathological diagnosis of prostatic adenocarcinoma, Gleason score 10 (Grade Group 5), with signet-ring-cell-like features, was made.

**Figure 3:**
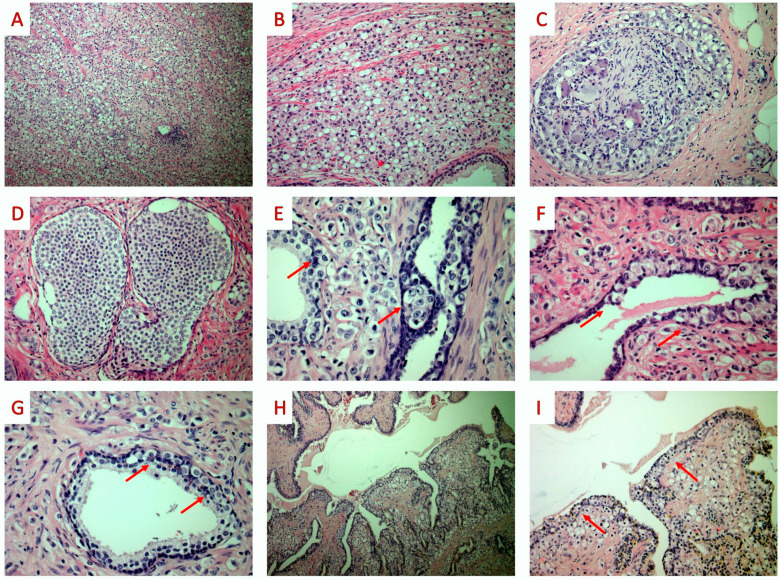
Radical prostatectomy: histopathologic findings. (**A**) On low-power examination, the prostate showed diffuse infiltration by solid nests of poorly-differentiated tumor cells (H&E; ×40). (**B**) On higher-power examination, several tumor cells displayed signet ring cell morphology (H&E; ×100). (**C**) Perineural invasion was commonly seen (H&E; ×200). (**D**) The invasive carcinoma was associated with intraductal carcinoma (H&E; ×200). (**E**–**G**) Pagetoid spread was observed in adjacent prostatic acini and ducts (red arrows) (H&E; ×200). (**H**) Seminal vesicles were infiltrated by the tumor (H&E; ×40). (**I**) On medium-power examination, the seminal vesicles were also involved by pagetoid spread (red arrows) (H&E; ×100) (original, previously unpublished photos).

Since signet ring cells were identified, a gastro-intestinal endoscopy and imaging studies (computed tomography scans, CT; magnetic resonance imaging, MRI; and positron emission tomography) were performed to exclude the possibility of a metastasis from a non-prostatic tumor as well as for an accurate tumor staging; all these exams did not reveal significant findings. A radical prostatectomy was performed. Histological examination revealed an extensive, bilateral prostatic infiltration by a high-grade adenocarcinoma. The tumor cells were arranged as a solid proliferation or as single, discohesive cells with signet ring cell morphology in more than 50% of the tumor volume. Foci of intraductal carcinoma were also intermixed with invasive carcinoma. Foci of perineural invasion were found, while lymphovascular invasion was not identified. The tumor showed focal unilateral (right-sided) extraprostatic extension and invasion of the wall of the seminal vesicles. The tumor showed limited (less than 3 mm) involvement of the ink-stained right anterior surgical margin. Pagetoid spread of tumor cells was identified in the surrounding prostatic acini and ducts, as well as in the epithelium of the seminal vesicles (Figure 3).

**Figure 4:**
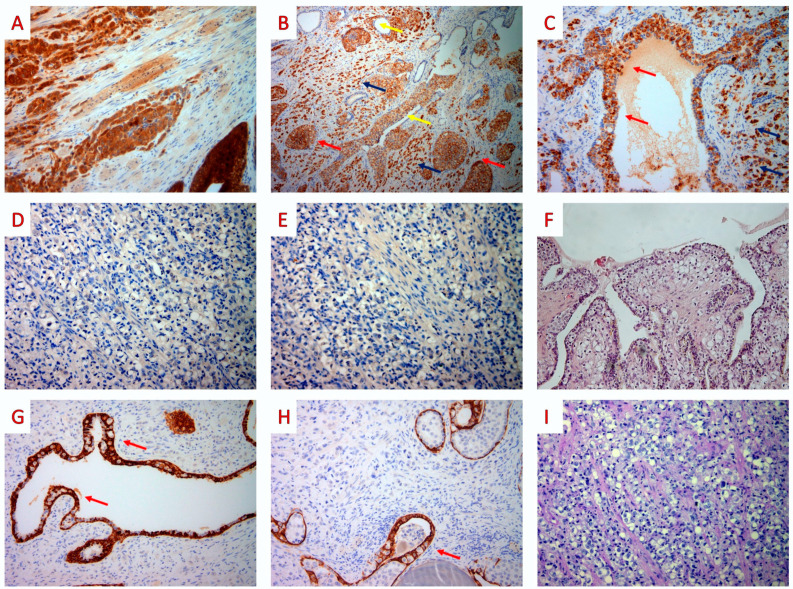
Radical prostatectomy: immunohistochemical and histochemical findings. (**A**) PSA showed uniform cytoplasmic staining in the tumor cells (PSA mouse monoclonal ER-PR8, Zytomed × 100). (**B**) Tumor cells displayed diffuse cytoplasmic staining for P504S in the invasive component (blue arrows), in the intraductal carcinoma (red arrows), and in the pagetoid tumor cells (yellow arrows) (AMACR rabbit monoclonal 13H4, DAKO × 40). (**C**) The invasive component (blue arrows), the intraductal carcinoma (not shown), and the pagetoid tumor cells (red arrows) stained for P504S (AMACR rabbit monoclonal 13H4, DAKO × 200). (**D**–**F**) Staining for CK7, CK20, and CDX-2 were negative (cytokeratin-7 mouse monoclonal OV-TL, Zytomed, cytokeratin-20 mouse monoclonal Ks20.8, DAKO, CDX2 rabbit monoclonal EPR2764Y, Zytomed × 100). (**G**,**H**) The basal cells of ducts and acini stained for CK34βE12 in contrast to neoplastic pagetoid cells (red arrows) (**G**): HMWCK mouse monoclonal 34bE12, Zytomed × 100; (**H**) HMWCK mouse monoclonal 34bE12, Zytomed × 40). (**I**) Tumor cells did not stain for PAS–Alcian blue (DAKO ARTISAN special staining kit × 100) (original, previously unpublished photos).

Due to the rarity of primary prostatic SRCCs, an extensive immunohistochemical study was performed to exclude the possibility of metastatic disease to the prostate. The invasive carcinoma, IC, and tumor cells involved in PS expressed PSA and P504S, while CK7, CK20, CDX-2, and GATA-3 were negative. CK34βE12 was expressed by the basal cells of ducts and acini, while it was negative in the tumor cells. Finally, on histochemical analysis, the tumor cells did not stain for periodic acid–Schiff (PAS)/Alcian blue (DAKO ARTISAN special staining kit) (Figure 4).

**Figure 5:**
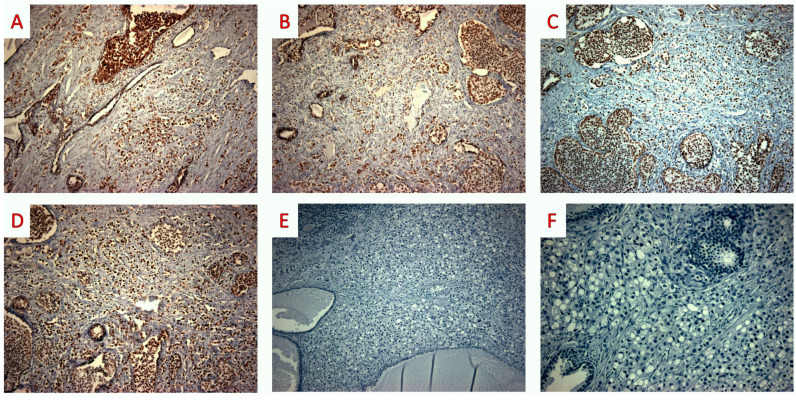
Radical prostatectomy: immunohistochemical findings. (**A**–**D**) Tumor cells displayed positive staining for the mismatch repair system proteins: MLH1 ((**A**) MLH1 mouse monoclonal ES05, DAKO × 100); PMS2 ((**B**) PMS2 rabbit monoclonal EP51, DAKO × 100); MSH2 ((**C**) MSH2 mouse monoclonal FE11, DAKO × 100); and MSH6 ((**D**) MSH6 rabbit monoclonal EP49, DAKO × 100). (**E**,**F**) PD-L1 staining was negative in tumor cells ((**E**) PD-L1 mouse monoclonal 22C3, DAKO × 100); ((**F**) PD-L1 mouse monoclonal 22C3, DAKO × 200) (original, previously unpublished photos).

Based on the above findings, we diagnosed an SRCC of the prostate, with a Gleason score of 10 (Grade Group 5), pagetoid spread, and associated foci of intraductal carcinoma, and a pT3b stage was assigned. We also performed immunohistochemical testing for the MMR proteins MLH1 (mouse monoclonal ES05, DAKO), PMS2 (rabbit monoclonal EP51, DAKO), MSH2 (mouse monoclonal FE11, DAKO), and MSH6 (rabbit monoclonal EP49, DAKO), and for PD-L1 (PD-L1 mouse monoclonal 22C3, DAKO). Intense nuclear staining was present in the majority of tumor cells (invasive and intraductal carcinoma, as well as tumor cells showing pagetoid spread) for all four of the MMR proteins. PD-L1 staining was uniformly negative in tumor cells (invasive carcinoma, IDC, or PS areas) with focal and weak to moderate expression in less than 3% of inflammatory cells (Figure 5).

The patient was treated with gonadotropin-releasing hormones, anti-androgens (Verodex and Leuprorelin), and radiotherapy. Eighty-eight months after surgery, the patient is alive with no evidence of biochemical or radiological recurrence.

## 4. Discussion

According to the latest (fifth) edition of the classification of urinary and male genital tumors of the World Health Organization (WHO), acinar adenocarcinoma is the most frequent prostatic malignancy, but other unusual subtypes (such as the SRCC, sarcomatoid, pleomorphic giant cell, or PIN-like types) may also occur as pure or combined forms [35]. In particular, the WHO accepted the suggestion of Guerin et al. to consider SRCC as a subtype of high-grade PA and not as an unrelated special histotype [36].

SRCC of the prostate is a vanishingly rare entity; to our knowledge, around 200 cases have been reported in the English literature. Patient age ranges from 50 to 85 years, with a median age of around 68 years [5]. Clinically, at the time of diagnosis, most patients (around 75%) have locally advanced or metastatic disease [9,37]. The presenting symptoms are similar to the typical high-grade PA, such as frequent urination, dysuria, hematuria, and pelvic pain [38]. Additionally, symptoms related to metastases may appear [8]. The patients may have variable serum PSA values ranging from 0.19 ng/mL to 1990 ng/mL [2]. Radiological findings (multiparametric MRI, transurethral ultrasound) are not specific [38].

The diagnosis of primary SRCC is mainly based on pathological findings. Histologically, SRCC is characterized by the presence of cells with a signet ring cell appearance that results from nuclear compression to the periphery. According to the WHO classification, the prostatic signet ring cells usually stain negative for mucins, supporting the use of “signet-ring cell-like” terminology [35], while other studies found that staining for PAS, Alcian blue, and mucicarmine could be positive in 60%, 60%, and 40% of SRCC cases, respectively [4]. In our case, the PAS–Alcian blue staining was negative.

Epstein and Lieberman have proposed that the SRCC diagnosis requires that at least 25% of the tumor volume be signet-ring cells, while other studies did not follow this criterion [37]. The latest (fifth) edition of the WHO classification of urinary and male genital tumors confirmed that the signet ring cells should represent at least 25% of the tumor volume [35]. SRCCs totally composed of signet-ring cells are exceptional, while a component of acinar PA is usually evident in some areas [35].

Immunohistochemically, PSA and PSAP are positive in 87% of cases in primary prostatic SRCCs, thus supporting a prostatic origin of the tumor [9]. However, high-grade and/or metastatic PAs may lose PSA reactivity [39]. CDX-2 immunostaining may show patchy and focal expression in a small proportion of primary SRCCs of the prostate, usually being more diffuse in gastrointestinal adenocarcinomas [40,41]. GATA-3 is a reliable marker to distinguish urothelial from prostatic carcinomas, typically resulting negative in PAs [42]. In most cases of SRCC, CEA is negative [38]. Additionally, the high expression of estrogen receptor-beta and Ki-67 is related to a poorer prognosis [43]. In needle core biopsies, smooth muscle cells and lymphocytes could appear similar to the signet ring cells. In these cases, the IHC panel should include SMA and LCA, which are typically not expressed by PA [6]. Ultrastructural studies have revealed that in prostatic SRCCs, the signet-ring cell morphology results from vacuoles or intracytoplasmic lumina [44].

To establish the diagnosis of a primary prostatic SRCC, it is also essential to perform upper gastric endoscopy, colonoscopy, cystoscopy, and abdominal computed CT to exclude metastatic involvement of the prostate by tumors arising from the gastrointestinal tract, urinary bladder, or pancreas [2]. Since the therapeutic approach is different, it is crucial to distinguish between these entities [9]. In our case, the tumor cells displayed uniform staining for PSA and PSAP, and they were negative for GATA-3, CDX-2, CK7, and CK20. Additionally, imaging and endoscopy exams ruled out the possibility of metastasis.

The IHC study also assisted us in the diagnosis of PS by the tumor cells. PS is a very rare phenomenon in PA, either in prostatectomies or in distant metastases such as skin or penile mucosa, which sometimes mimics melanomas or other primaries [45,46,47].

The differential diagnosis of PS from PA includes urothelial or colorectal carcinomas displaying PS to prostatic ducts and seminal vesicles [48,49,50] and intraepithelial pagetoid histiocytes [51]. In our case, the pagetoid intraepithelial cells showed high-grade atypia and expressed prostatic immunomarkers (PSA, PSAP, P504S), while the urothelial (GATA-3, CK34βE12), colorectal (CK20, CDX-2, CK7) and histiocytic (CD68) immunostains were negative.

IC is a complex, usually expansile intraductal proliferation of neoplastic cells arranged in solid or dense cribriform patterns (the latter of which shows more solid than luminal areas in >50% of the involved gland/duct); in the case of loose cribriform (<50% of the involved gland/duct) or micropapillary patterns, marked cytologic atypia and/or comedonecrosis should be present to define an intraductal proliferation as IC [35]. Urothelial carcinoma may mimic the solid pattern of IC, which is negative for GATA-3 and positive for PSA (as in our case). Frequently associated with an invasive high-grade PA (like in our case), IC should be identified by pathologists, especially in the context of prostatic core biopsies not showing invasive cancer or revealing a PA with a low Gleason score; indeed, the finding of IC alone may address patients to surgery [35]. The evidence of IC, either in biopsies or prostatectomy specimens, has been correlated to adverse histopathological and outcome features of the frequently associated invasive PA [35]. However, no recurrence was found after 88 months of follow-up in our case.

We found foci of IC showing a solid intraductal proliferation of atypical cells; although rare cases of high-grade PIN revealed signet ring cell features [52], such a pattern was not evident in our case. As IC is an entity recently introduced in the WHO classification, its incidence in the rarely reported prostatic SRCCs is unclear [35].

The treatment of SRCC is similar to that of the traditional adenocarcinomas of the prostate, which includes a variable combination of surgical procedures, hormonal therapy, adjuvant radiotherapy, and/or chemotherapy [37]. The response to hormonal treatment is unpredictable [9]. The highest survival was seen in patients who received a combination of surgical and hormonal therapy, while the shortest survival was described after surgical excision of the prostate alone [9]. Our patient was treated with prostatectomy followed by gonadotropin-releasing hormones, anti-androgens, and radiotherapy, showing a long-term (88 months) overall survival, as well as biochemical and radiological recurrence-free survival rates.

Despite the aggressive multimodal treatment, several studies have revealed that primary prostatic SRCC has a poor prognosis related to the frequently advanced local stage and higher rates of metastatic disease [53]. Warner et al. reported an average survival time of 29 months [9]. Interestingly, Wang et al. concluded that younger age is related to poor survival [54]. In the study by Saito and Iwaki, the three-year and five-year survival rates were 27.3% and 0%, respectively, while Fujita et al. reported a five-year survival rate of 11.7% [6,37]. In the study of Fujita et al., only the disease stage at the diagnosis was associated with survival [37].

On the other hand, some cases in the literature have shown a less aggressive clinical course (as for our patient). Gök et al. reported a case of an early-stage (pT2aN0) SRCC receiving hormone therapy and radiotherapy, which showed no evidence of disease after a follow-up of 16 months [6]. Two patients showed good responses to hormonal therapy and radiotherapy [55], reporting a survival period of 12 and 100 months, respectively [56,57], and one case had a good response to hormonal therapy alone with a follow-up time of 24 months [58]. Another patient showed a near-complete response to FOLFOX and Erbitux [59]. Finally, neoadjuvant hormonal therapy followed by radical prostatectomy has shown promising results in a few cases [55,60].

Concerning new targeted therapies, a subset of PAs that display high tumor mutation burden (TMB), PD-L1 expression, microsatellite instability, or MMR deficiency may respond to immunotherapy [21,22,23,24,25,26,27,28,29,30,31,32,33,34,61,62,63,64,65]. However, further studies should verify its role in SRCCs, as scant data are available in the literature. Molecular studies of prostatic SRCCs are very rare, probably due to the limited number of reported cases. Such studies could identify potential therapeutic targets similar to gastric and colorectal SRCCs [66].

The 2023 United States National Comprehensive Cancer Network (NCCN) guidelines [21] have allowed pembrolizumab administration after progression through prior docetaxel and novel hormone treatments in patients with metastatic castration-resistant PA (mCRPA) showing deficient MMR expression, high microsatellite instability (H-MSI) or TMB ≥ 10 mutations/Mb. The MSI/MMR status testing (either by molecular analysis or IHC) is recommended in mCRPA and is considered for castration-naïve metastatic PA. To the NCCN guidelines, PD-L1 IHC testing can be used to identify lung cancers most likely to respond to first-line anti-PD-1/PD-L1 inhibitors, while this evaluation seems unnecessary in PA; they did not report clear indications of the PD-L1 antibody clone to use for evaluating PA samples [21].

IHC is easy to use and cost-effective, but it may not predict tumor recurrence after radical prostatectomy; moreover, the MMR/MSI and PD-L1 status may not necessarily correlate to the immunotherapy response. Combined MMR/MSI and PD-L1 analysis was rarely performed in PA; we did not find previous relevant data about prostatic SRCC. For non-SRCCs, some authors favored an association between MMR loss and higher PD-L1 expression rates in tumor cells, but it is still difficult to understand the frequency and significance of MMR deficiency in PD-L1-positive and -negative groups [24,25,26].

A recent systematic literature review of PD-L1 expression in PA [63,64,65] reported the following:

(1) At least focal IHC expression of PD-L1 was found in the tumor cells of 29% acinar PCs (0–100% as to various studies), 7% ductal PAs, and 46% neuroendocrine carcinomas/tumors. Only 1 case of prostatic SRCC (reported by an abstract of Hashimoto et al.) was tested for PD-L1; however, the authors identified 2/110 (2%) PD-L1+ PAs in a series including 105 acinar, 4 ductal, and 1 SRCC PA, but they did not clearly report which histotypes resulted positive [67].
(2) About one-third of the tested PAs (280/883, 32%) revealed MSI (210/263, 80%) or MMR IHC loss; at least 1, ≥2, ≥3, or 4 MMR proteins were lost by IHC in 12%, 4%, 1% and 0.4% of PA cases, respectively. MSH6 was the most frequently lost (45/60, 75%), followed by PMS2 (29/60, 48%), MSH2 (11/60, 18%), and MLH1 (3/60, 5%).

As to these results and to our present systematic literature review, no clear data about MMR or microsatellite testing in prostatic SRCCs seem to have been previously reported; our case retained the expression of the four MMR proteins.

In addition to the unclear data of Hashimoto et al. [67], our present systematic literature review resulted in only one paper describing four prostatic SRCCs tested for PD-L1 (Table 1) [2].

Including our case, the SRCC patients tested for PD-L1 showed an age range of 63–75 (mean 68.8) years and serum PSA levels ranging from 10 to over 100 ng/mL (mean 48.4 ng/mL) [2]. These men were variably treated for PAs, usually revealing a high grade group (4–5) except for 1 case; information about tumor stage was sometimes missing, but only 1 patient seemed to clearly show metastatic disease (bone), and all patients were alive 20 to 142 months after diagnosis (mean follow-up: 94.6 months) [2].

Teng et al. [2] tested the PD-L1 clone 28-8 (rabbit monoclonal, dilution 2 μg/mL; ab205921, Abcam, Cambridge, UK) on their four cases of SRCC, focusing on the evaluation of the stromal expression. The authors multiplied a non-completely clear “score for the percentage by the intensity score” (0: absent staining; 1: weak; 2: moderate; 3: strong); they found a higher expression of PD-L1 (score 4/4 for all 4 cases) and histiocytic markers (CD163, CD68) in primary SRCC specimens than in either adjacent non-tumoral prostatic tissue or in other non-SRCC PC samples showing variable Gleason scores; no differences concerning the staining distribution of PD-1, CD4, and CD8 expression were identified. They suggested that tumor-associated macrophages may inhibit the T-cell function, promoting tumor progression. In their study, it seems that “many tumor and immune cells” expressed PD-L1, but the exact percentage of positive tumor, immune, and/or stromal cells was not completely clear for each case. We have chosen clone 22C3, which is the most used for pembrolizumab eligibility [21]; we found that PD-L1 did not show staining of tumor cells in the invasive carcinoma, IDC, or in cells displaying PS.

PD-L1 expression by tumor cells may correlate to prognostically adverse clinical–pathological features, such as a high grade group, an advanced tumor stage (high pT, lymph node/distant metastases), positive surgical margins, and castration resistance [63,64,65]. However, some results are controversial, and the PD-L1 IHC evaluation in PA can be influenced by various biases. First, variable scoring systems were used in different studies, assessing expression only in tumor cells or also in inflammatory cells (combined positive score, CPS; tumor proportion score, TPS; semiquantitative analysis, percentage of positive cells × staining intensity, etc.) [63,64,65]. Moreover, according to a literature review, different cut-offs for positivity were applied (≥1% of tumor cells: 26% of PAs; ≥5%: 9%; >50%: 2%; TPS: 18%; CPS ≥ 1: 78%) [63,64,65]. In addition, other interfering factors included inter-observer variability in the interpretation of the IHC slides, different antibody clones and sample types (metastatic vs. primary tumors, radical prostatectomies, biopsies, autopsies, tissue microarrays), tumor heterogeneity, and other pre-analytical variables (fixation, decalcification, etc.) [63,64,65]. In a previous literature review [63,64,65], various antibody clones were tested in multiple samples (22C3, SP263, PA5-18337, 28-8, E1L3N, ABM4E54, SP142, 5H1, etc.), showing a positivity rate in tumor cells ranging from 3% to 50%. Clone 22C3 (the one we used in our case) seems to be the second most frequently tested antibody in PA after clone E1L3N (positivity rate: 35%), being expressed in 11–41% of PA cases (depending on different scoring systems). Clones 28-8, SP263, and SP142 resulted in lower positivity rates (15%, 6%, and 3%, respectively), while other clones were tested in less than 200 cases [63,64,65].

## 5. Conclusions

In summary, we have reported a case of a prostatic SRCC (stage pT3b, Grade Group 5) showing PS to prostatic ducts and seminal vesicles and foci of IC, being associated with long-term survival. Diagnosing SRCC of the prostate can be difficult, requiring a multidisciplinary approach (clinical, endoscopic, radiologic, and histopathological). Its clinical behavior is aggressive, with most patients presenting with locally advanced or metastatic disease; however, long-term survivors may occur (as in our case). There are no established guidelines for treating this subtype differently from other high-grade PAs. However, due to its ominous prognosis, the use of an aggressive therapy scheme seems reasonable. Further investigations, including molecular studies and MMR/PD-L1 evaluation, are needed to better understand this rare entity.

## Figures and Tables

**Table 1:** Details of the prostatic SRCC cases tested for PD-L1 according to our systematic literature review.

Authors	Age (Years)	PSA (°) (ng/mL)	Symptoms	GG	Stage	Treatment	FU (mo)
Teng et al., 2022: case 1 [2]	75	49.73	NR	3	T2cNxM0	biopsy + HT (FLU)	NED, 117
Teng et al., 2022: case 2 [2]	70	10.09	hematuria (2 weeks)	4	pT1NxMx (*)	biopsy + HT (BIC + ZOL) + RP	NED, 106
Teng et al., 2022: case 3 [2]	69	>100	hydronephrosis	5	M1 (bone)	biopsy + HT (FLU + ZOL + zoledronic acid) + TURP + BO + Cht (DOC)/ZOL	AWD, 142
Teng et al., 2022: case 4 [2]	67	65.87	difficult urination (1 week)	5	NR	biopsy	AWD, 20
Our case	63	16.39	acute urinary retention, hematuria	5	pT3b cN0 cM0	biopsy + RT + HT (Gn-RH, VER, LEU) + RT	NED, 88

(°)—at presentation; (*)—potential bone metastasis; AWD—alive with disease; BIC—bicalutamide, oral, 50 mg, 1 per die; BO—bilateral orchiectomy; Cht—chemotherapy; DOC—docetaxel, intravenous, 120 mg/dL for 21 days; FLU—flutamide, oral, 250 mg, 3 per die; FU—follow-up; GG—grade group according to the WHO classification [35]; Gn-RH—gonadotropin-releasing hormones; HT—hormonal therapy; LEU—leuprorelin; mo—months; NED—no evidence of disease; NR—not reported; RP—radical prostatectomy; RT—radiotherapy; TURP—transurethral prostatic resection; VER—Verodex; ZOL—Zoladex, subcutaneous, 3.6 mg, 1 per month.

## Data Availability

Not applicable.
